# A health in all policies approach to promote active, healthy lifestyle in Israel

**DOI:** 10.1186/2045-4015-2-16

**Published:** 2013-04-22

**Authors:** Yannai Kranzler, Nadav Davidovich, Yonina Fleischman, Itamar Grotto, Daniel S Moran, Ruth Weinstein

**Affiliations:** 1 Public Health Services, Israel Ministry of Health, 39 Yirmiyahu Street, Jerusalem, Israel; 2 Faculty of Health Sciences, Ben Gurion University of the Negev, Beer Sheva, Israel; 3 Faculty of Health Sciences at the Ariel University Center of Samaria, Ariel, Israel

**Keywords:** Health in all policies, Obesity, Intersectoral action, Health governance

## Abstract

In December 2011, Israel launched the National Program to Promote Active, Healthy Lifestyle, an inter-ministerial, intersectoral effort to address obesity and its contribution to the country’s burden of chronic disease. This paper explores the National Program according to the “Health in All Policies” (HiAP) strategy for health governance, designed to engage social determinants of health and curb health challenges at the causal level. Our objective is twofold: to identify where Israel’s National Program both echoes and falls short of Health in All Policies, and to assess how the National Program can be utilized to enrich the Health in All Policies research-base.

We review Health in All Policies’ evolution, why it developed and how it is diverges from other approaches to intersectoriality in health. We describe why obesity and related chronic diseases necessitate an intersectoral response, cite obstacles and gaps to implementation and list examples of HiAP-type initiatives from around the world. We then analyze Israel’s National Program as it relates to Health in All Policies, and propose directions through which the initiative may constitute a useful case study.

We contend that joint planning, implementation and to a limited extent, budgeting, between the Ministries of Health, Education and Culture and Sport reflect an HiAP-approach, as does integrating health into the policymaking of other ministries. To further incorporate health in all Israeli policies, we suggest leveraging the Health Ministry’s presence on governmental and non-governmental committees in areas like building, land-use and urban planning, institutional food policy and environmental health, and focusing on knowledge translation according to the policy needs, strengths and limitations of other sectors. Finally, we suggest studying the National Program’s financing, decision-making and evaluation mechanisms in order to complement existing research on the implementation of Health in All Policies and intersectoral action for health.

## Introduction

Intersectoriality and health-promoting public policy are gaining prominence as strategies for sustainably and equitably fostering population-wide health [1-6]. At least 16 countries, the European Union and the World Health Organization (WHO) have adopted “Health in All Policies” (HiAP) [7], a “policy practice of including, integrating or internalizing health in other policies that shape or influence the social determinants of health” [[8], p.12].

Obesity, characterized by complex determinants, multi-level stakeholders, concentrated morbidity and mortality among lower-income groups and inequitable access to solutions, is a test-case for HiAP. According to some, it is the quintessential 21^st^ century public health crisis [3]. In December 2011, Israel launched the National Program to Promote Active, Healthy Lifestyle, an inter-ministerial, intersectoral effort to address obesity and its contribution to the country’s burden of chronic disease. The aims of this paper are twofold: to analyze Israel’s National Program according to HiAP’s theoretical framework, and to assess how the program can be studied to enrich HiAP’s research base. How does the National Program echo HiAP? Where does it fall short? How can Israel’s experience deepen the public health community’s understanding of HiAP-type intersectoral collaboration?

We begin with an overview of HiAP’s evolution. Why did it develop? How does it diverge from other approaches to intersectoriality in health? Has it been implemented? We then describe why obesity necessitates an intersectoral response and identify implementation obstacles and research gaps. Next, we present Israel’s National Program, describe where it incorporates HiAP and suggest ways to further strengthen Israel’s intersectoral potential. Finally, we propose the program as a case study in HiAP, suggesting research opportunities that the program presents.

### The imperative to reach beyond health

The social determinants of health are well documented; housing conditions, education, income and access to safe, meaningful employment weigh heavily on longevity and health status [4,9,10]. As such, many public health scholars now advocate engaging social and economic policy arenas [4,7]. Intersectoriality has become uniquely relevant, as economic strain clashes with health systems’ struggle to meet the treatment needs of increasingly obese, chronically ill and ageing populations [4]. Addressing determinants allows governments to lower a population’s mean level of risk, facilitating a more comprehensive impact than downstream interventions where responsibility falls primarily on the health sector [11].

Crossing sectors has been a cornerstone of health promotion since the field gained prominence in the second half of the 20^th^ century. The Alma Atta declaration in 1978 defined health as a “social goal whose realization requires the action of many other social and economic sectors in addition to the health sector.” The Ottawa Charter on Health Promotion of 1986 called for “healthy”, or health-promoting public policy and supportive environments and introduced health promoters as “brokers” between sectors. The 1997 WHO Conference on Intersectoral Action for Health urged health authorities to form working relationships with other sectors. In 2005, the WHO’s Commission on the Social Determinants of Health recommended health-promoting policies in education, industrial affairs, taxation and welfare [3].

### From healthy public policies to health in all policies

In 2010, the Adelaide Statement on Health in all Policies outlined the need for “a new social contract between all sectors to advance human development, sustainability and equity, as well as to improve health outcomes” [12]. Whereas the previous model of healthy public policies involved advocating for other sectors to adopt specific health-promoting measures, the Aidelaide Statement articulated a networking strategy. In HiAP, joint policymaking would allow stakeholders to address issues critical to all members of “the network.” “The balance,” Kickbusch explains, “appears to be shifting from ‘intersectoral action for health to intersectoral action for shared societal goals” [[3], p.19].

Health, in HiAP, has the potential to be a tangible proxy for equity and societal wellbeing. It is a human right to protect, a public good to enable, and an economic resource in which to invest [4]. HiAP demands that the health sector becomes acquainted with other sectors’ policy goals and processes in order to steer policymaking in health-promoting directions and foster a governmental agenda that is congruent with and complimentary toward health goals [4,7,13]. Instead of competing for health to be placed at the center of an increasingly complex, expensive and saturated policymaking agenda, HiAP advocates leveraging health in the service of other agendas [3].

HiAP is anchored in formal governmental structures and mechanisms. Goals include addressing supra-governmental trends and agendas like distributions of power, money and resources, education for all, gender equity, urban planning, fair financing and market accountability [4,7,13]. It extends beyond information-sharing between sectors, and focuses on integration in policy development, budget-management and implementation [7]. Governance occurs horizontally (across similar-level government agencies) and vertically (from high-level to street level government). Like many healthy public policies, HiAP targets society as a whole. Instead of sufficing to increase healthcare for vulnerable groups, HiAP’s aim is to curb health inequalities at the causal level [7].

### Implementation gaps, research opportunities

Finland pioneered intersectoriality in the 1970s and formally introduced HiAP in 2006 [4]. Countries around the world are currently attempting HiAP-type strategies to address issues like obesity, food safety and tobacco. We describe several examples in Appendix 1. Few cases, though, have been researched in-depth [7], and full scale implementation remains limited [3,7,9,13].

Shankardass et al [7] found that 75% of so-called HiAP initiatives focused on increasing access to health services. Less than a third applied “upstream” interventions, like addressing income or power redistribution. According to Bacigalupe et al [14], few governments have applied health-promoting public policies on a system-wide level. It has been difficult to cause societal actors outside the health sector to “think health” and, thus, to foster it [3]. Several challenges have arisen:

HiAP necessitates working alongside sectors with their own priorities and over whom health authorities have no control [13]. HiAP can appear threatening, like health sector imperialism [4]. Health implications tend to manifest subtly and slowly, making them easily misunderstood or overlooked, as well as limiting their political value [3,13,14]. Many neo-liberal 21^st^ century public institutions balk at intervening in social and economic spheres within which determinants of health are found [14].

Implementation gaps lead to research questions: To what extent, when and why is HiAP viable? Under what circumstances do policymakers in other sectors embrace public health? What benefits do they perceive in engaging in health promotion? What barriers do they face? Are there solutions they can propose, that members of the health sector have not, or cannot? Which governance mechanisms determine the most effective actions? What are health promotions’ political assets and liabilities?

Shankardass et al’s review [7] found that case studies on intersectoral collaboration are rarely analytical, tend towards superficiality, and do not address the perspectives of policy entrepreneurs, the health sector and other stakeholders. Further study is required to understand collaborative mechanisms, how to approach other sectors, communication strategies, budget management, decision-making and the importance of economic impacts to each sector. Walls et al [15] cite the need to characterize political contexts conducive to intersectoriality. McKinnon et al [16] suggest utilizing current efforts as natural experiments. It is necessary to continue identifying the economic strain caused by health challenges like obesity [16], but each stakeholder has its own economic priorities, the nuances of which must be ascertained.

In light of the research gaps, existing HiAP-like initiatives are critical opportunities for study. In the following section, we will describe the obesity epidemic and describe why many believe that obesity represents a “test case” for HiAP [3]. We will then present and analyze Israel’s National Program to Promote Active, Healthy Lifestyle.

### Obesity: a test case for health in all policies

Global and national health organizations sharpened the discourse around a “global obesity epidemic” at the turn of the 21^st^’ century [6,17]. Consequences include increased morbidity and mortality, expenditures of up to 8% of countries’ healthcare costs [18] and soaring indirect costs [16]. Intersectoral approaches like HiAP are often invoked in response to the magnitude of the societal burden obesity creates and the social determinants from which it is often a result [3].

Obesity is rooted primarily in lifestyle: Refined foods, snacking, sugary drinks, meals away from home, increased consumption of saturated fats and exaggerated portion sizes, alongside motorized transportation and increasingly sedentary behavior at home, school and work [6,19,20]. Because obesity reflects an unhealthy lifestyle, responses initially focused on educating toward a healthy lifestyle, through, for example, health education campaigns, short term projects and sporting events [6]. But as low socio-economic groups bear the brunt of higher concentrations of obesity [19], criticism has grown against the focus on individual behavioral change, which ignores the unequal distribution of the determinants of obesity and inequitable access to solutions [6,18,19]. Framing lifestyle as a function of choice, critics argue, inadvertently discriminates against individuals whose choices are limited.

Low socio-economic groups have an increased risk of becoming obese for several reasons. Smaller activity spaces and transportation constraints foster health-discouraging environments [21]. A prevalence of inexpensive high-calorie foods and infrequent exposure to healthy alternatives breed health-discouraging food environments [16,19]. Insecure and inflexible employment conditions and unsupportive social networks result in weight gaps often reflecting social gaps [19]. Friel et al [19] describe an additional disadvantage to focusing on individual behavior: Wealthier individuals may make requisite changes. Poorer ones, more likely, will not. This threatens to further widen gaps, as obesity leads to decreased household incomes, earlier retirement and higher dependence on state benefits.

### Addressing obesity “in all policies”

The social determinants of obesity intersect multiple levels and policy terrains, including agriculture, manufacturing, education and trade [6,22]. Engaging these sectors is, therefore, crucial [1,3-7,9,15,16]. Kickbusch [3] argues that obesity is the type of complex problem that necessitates HiAP. Others, while not citing HiAP, have pointed to the need for systematic intersectoriality in order to address obesity [1,5,6,18]. The WHO, as well, recommends adopting a systematic approach to combating obesity and chronic disease. Several countries are attempting to do so [1,7], pursuing fruit and vegetable subsidies, taxes on sugary drinks and/or trans fats, marketing-bans on unhealthy foods to children, removing junk food from schools and increasing access to physical activity. These structural changes are seen as less susceptible to unequal uptake between high and low socio-economic groups [15].

Despite the call for systematic, intersectoral action, analyses of obesity prevention policy note obstacles similar to the challenges associated with implementing HiAP. For example, a systematic approach demands the political will to form alliances across bureaucratic divides [6]. It entails health workers leaving their professional comfort zones to try policy instruments with which they are unfamiliar [15,16]. Addressing the food system includes clashing with a powerful food industry that often succeeds at framing regulations as government infringing upon personal freedom [6]. Slow change limits political attractiveness [22].

### Obesity and chronic disease in Israel

Fifteen percent of Israel’s adult population is obese; roughly one out of two is overweight. More than one out of five children aged 12-18 are overweight or obese [23]. Overweight/obesity levels have increased steadily over the last four decades [24], more drastically among individuals of lower socio-economic status [24,25].

Obesity and overweight account for approximately 3,105 deaths per year, 7.7% of all yearly deaths [26]. The rate of mortality due to diabetes in Israel is twice as high as the average in Western countries [23], with the Arab population suffering in particular [27]. In 2007, for example, mortality attributed to diabetes was approximately 61% for Arab men and 27% for Jewish men [27].

Since 1994, hypertension levels have increased by more than 250%. Diabetes levels have doubled since the 1950s and are expected to double-triple again in the next 20 years, barring a radical change in nutrition and exercise patterns [23]. Treatment of morbidity due to obesity and overweight in Israel costs an estimated 1.92 billion shekels, with an additional 1.89 billion shekels in productivity losses and 1.95 billion in other indirect costs [26].

Only 32% of Israelis ages 21 and over engage in the recommended amount of physical activity (34.6% among Jews/21.6% among Arabs, 36.3% among men/28.8% among women). Individuals with greater income and higher education are more physically active than those of lower socio-economic status [28]. In the Arab sector, only 14.7% of adolescents exercise regularly [29]. According to the most recent “Health Behaviors in School-Aged Children (HBSC)” cross-national survey, Israel has the second highest rate of children ages 11, 13, and 15 who had not engaged in physical activity during the previous 7 days (12.3%). The same study revealed that Israel has the highest rates of children ages 11, 13, and 15 who play computer games for more than 4 hours per day (28.5%) [30].

All segments of the population consume substantial amounts of sweetened drinks every day, especially Arab men (65.1%) and Arab women (42.6%), according to one sample [31]. Caloric intake in Israel has increased consistently since the 1970s, and in 2007, surpassed the European average [29]. According to one sample, whole grain products are consumed in just half of the homes in Israel [32]. Israelis’ average salt intake is more than double the recommended level [29]. While Israelis, in the past, have consumed adequate amounts of fruits and vegetables, recent evidence from the Ministry of Agriculture suggests a decrease, especially among low income groups [33].

### The national program to promote active, healthy lifestyle

In December, 2011, the Social and Economic Affairs Cabinet accepted a resolution outlining a National Program to Promote Active, Healthy Lifestyle, aimed at curbing obesity and the rise in chronic disease. Initiated by the Health Ministry, it is a government-wide effort, led by the Ministries of Health, Education, and Culture and Sport. The Ministries of Finance, Agriculture, Industry, Trade and Labor, Transportation, Communications, Environmental Protection, as well as local governments, the private sector, NGOs and civil society have signed on, as well.

### Program development process

The National Program developed in stages. First, the obesity, nutrition and physical activity working groups of the “Healthy Israel 2020” project recommended goals and policy guidelines [29]. Second, mid-level members of the Ministries of Health, Education and Culture and Sport translated the “2020” recommendations into an operative agenda that met the needs of the three ministries, including policy initiatives, budgetary arrangements, and division of labor. The directorship of the Health Ministry instructed ministry workers to ensure leadership roles for the other ministries involved. Third, the Ministry of Health obtained agreements to collaborate from additional ministries. A staff with representatives from the Ministries of Health, Education and Culture and Sport as well as an independent staff representing the three ministries finalized an acceptable - and passable – government resolution. Finally, the government adopted the resolution, which outlined policies and budget commitments from the Ministries of Health, Education, Culture and Sport, Finance and Agriculture.

### Program goals

The National Program’s quantitative targets include increasing the number of Israelis at a healthy weight by 10%, decreasing childhood and adolescent obesity by 20%, adult obesity by 10% among Jewish Israelis and, due to higher current obesity rates, by 15% in the Arab sector.

Behavioral goals include raising the number of individuals engaging in recommended physical activity levels by 20% among Jewish boys and 25% among Jewish girls and Arab boys and girls, and among adults by 20% for Jewish men, 25% among Arab men and Jewish women and 30% among Arab women. Additional targets include decreasing the number of children who watch at least two hours of television daily by 20%, decreasing daily salt consumption per person from nine grams to six and consumption of junk foods (as defined by the department of nutrition) by 20%. Additional targets are being set to increase whole grain-intake and breastfeeding rates.

### Program components

The National Program emphasizes three strategies: increasing knowledge, fostering health-promoting environments and incentivizing organizations and municipalities to engage in health promotion. The following are the programs’ primary components, in order to implement these strategies in the main settings which comprise Israeli life.

### Intersectoral committees and working groups

Intersectoral working groups identify entry points, ensure a diverse knowledge base and combine projects and budgets when priorities overlap. Leadership is provided by an intergovernmental steering committee led by the Health Ministry’s Director General, with representation from the Ministries of Finance, Agriculture, Interior, Trade, Labor and Industry and Communications, HMOs, the Israel Defense Forces and others. The government resolution defined two committees: The first, to devise strategies to integrate health promotion in workplaces, and the second, to identify regulatory measures to encourage consumption of healthy foods. The triministerial committee that developed the program has continued to lead its implementation.

### Legislative agenda

Legislative goals include removing junk foods from schools, taxing unhealthy foods like soda and/or trans fats, providing tax breaks on workplace purchases of healthy refreshments, lifting the requirement to obtain a doctor’s permission to join a health club, banning advertisements of unhealthy foods during children’s television programming, requiring restaurants to label menus with calorie contents and mandating front-of-package food labeling.

As of the beginning of 2013, the law banning unhealthy foods from schools passed its first parliamentary reading, and is likely to pass the required second and third readings following the elections. The Tax Authority authorized the tax break on healthy workplace purchases. Front-of-package and simplified food labeling regulations will take effect in 2013.

The Ministry of Health continues to work with the Ministry of Communications as well as television and cable authorities and other stakeholders to update ethical codes to ban the marketing of unhealthy foods during children’s programs and require that commercials for unhealthy foods during general viewing hours include a visual message which warns viewers of high fat, trans fat, sugar and/or salt content. Regulations will include guidelines regarding permitted ways to advertise foods, such as banning manipulative practices like marketing via famous personalities.

Drafts of the laws removing the requirement to obtain a doctor’s permission in order to join a gym and requiring calorie-labeling at restaurants are being finalized by the Ministry of Health and the relevant ministries. The Ministries of Health and Finance continue to work together to identify sustainable economic interventions to make healthy foods more accessible and/or unhealthy foods more expensive. One current proposal is to tax imported sugar, a primary ingredient in processed foods.

### The education system

The Ministry of Education declared 2011-12 to be “The Year of Active, Healthy Lifestyle.” Splitting the initiative’s funding, the Ministries of Education and Health trained teachers and principals to turn schools into health-promoting environments and appointed school, regional and national councils of health-promoting students. Over the course of the school year, 85 schools gained accreditation as health-promoting schools. Another 100 will be recognized in 2012-13. As follow-up, the Ministry of Education added health promotion to its list of educational objectives, and launched a fruit and vegetable scheme with the Health and Agricultural Ministries, modeled after similar programs in the European Union and the United States.

### Municipalities

“Municipalities Promoting Active, Healthy Lifestyle” began as a program of the Ministry of Culture and Sport. The Ministries of Health and Education joined in 2011 and together with the Ministry of Culture and Sport now support 15 municipalities, chosen according to location, population-size, ethnicity and socio-economic status, with a focus on reaching disadvantaged and marginalized populations. In addition to funding, the program includes guidance in program-design and implementation. Requirements from the municipalities include their own budget commitment, a designated project manager, a mayor-led steering committee, needs assessment, policy and environmental change at public institutions, special focus on weak and special-needs populations, proof of sustainability and evaluation. The Education Ministry outlined requirements for schools under each municipality’s jurisdiction and will provide the professional infrastructure to ensure that these requirements are met.

In partnership with the Center for Local Government, the National Program is also strengthening Israel’s Healthy Cities Network, created in 1990 [34], by adding cities and funding local and national staff. Additionally, following the National Program’s launch and after revealing in a survey that several municipalities’ public parks and exercise facilities are closed to informal/spontaneous physical activity, the Ministry of Culture and Sport directed municipalities to open all facilities. Municipalities’ Departments of Sport are identifying solutions that allow citizens access to public courts, fields and gymnasiums without compromising security and maintenance.

### Communities

Incentives for Israel’s four health providers, whose responsibilities include delivering primary care, include rewards for hiring health promoters and providing programs for diabetes and/or overweight patients and guidance for overweight children and their parents. Authorized by the Prime Minister in April 2012 and funded by the Ministry of Finance through an expansion of the national health budget, grants are 50% higher for interventions in socially and/or geographically marginalized communities.

In addition, the Health Ministry has oriented its district offices’ health promotion programs to focus on active, healthy lifestyle, including programs like health-promoting nursery schools and women’s walking groups. Finally, NGO-led activities supported through the National Program include active transport (walking or biking) to school, health-promoting dormitories for at-risk youth, edible gardens in nursery schools and providing bicycles and training to disadvantaged youth.

### Social marketing

The National Program is launching a social marketing program in partnership with Tel Aviv University, which boasts Israel’s leading social marketing team. In order to communicate effectively with subgroups and address their barriers and benefits related to active, healthy lifestyle, the initiative includes a social media-based effort as well as deploying community-based social marketers to areas participating in the “Municipalities” program.

### Other programs

The National Program includes supporting and expanding the following programs, which predate its launch:

The Ministry of Health is working with the food industry to voluntarily reduce salt content in processed foods, based on a successful model from England. Another program includes subsidizing fortification of flour in Israel’s Bedouin community, which suffers from malnutrition and high infant-mortality.

Interventions for children aged 0-3 focus primarily on protecting mothers’ ability to breastfeed at work, as well as training and certifying nurses at well-baby clinics as breastfeeding instructors. The nurses will also be given enrichment on how to educate parents to encourage their children to eat better and be more active. For older children, the Ministries of Health and Education created a website with games and other activities to get children excited about active, healthy lifestyle and worked with children’s TV networks to create health-related content. For adults, health promotion programs in large workplaces such as the military and the police provide healthier menus and both time and space for physical activity. The Health Ministry will be launching a pilot to promote health at workplaces in 2013.

### Evaluation

The National Program’s Evaluation Committee includes experts from Israel’s five universities as well as the Ministries of Health, Education and Culture and Sport. The committee informs the list of indicators and evaluation strategies, and will help draw conclusions. Grants will be available for universities and other research and evaluation institutions interested in researching and evaluating aspects of the program.

### Health in all Israeli policies?

The National Program represents a paradigm shift for the Ministry of Health, elevating health promotion from its status as a minor and often marginalized aspect of the ministry’s work, to the center of Israel’s health agenda. Table 1 illustrates how several aspects of the National Program echo Health in All Policies, in terms of the aforementioned WHO analytical framework.

**Table 1:** **The national program to promote active healthy lifestyle, according to the World health organization’s analytical framework for intersectoral governance**[7]

		**Governance actions**									**Example from Israel’s National Program to Promote Active, Healthy Lifestyle**
		**Evidence support**	**Setting goals & targets**	**Coordination**	**Advocacy**	**Monitoring & evaluation**	**Policy guidance**	**Financial support**	**Providing legal mandate**	**Implementation & management**	
**Intersectoral governance structures**	Ministerial linkages	√	√	√	√	√	√	√	√	√	The National Program, from leadership, to planning, financing, implementation and evaluation is a joint effort of the Ministries of Health, Education and Culture & Sport. The National Program’s staff is comprised of representatives from each ministry
	Cabinet committees and secretaries			√			√		√		The Government’s Economic and Social Affairs Cabinet adopted the National Program, incorporating the program’s policy guidelines, budget commitments and cross-ministerial coordination into the government’s social and economic agenda
	Public health ministers										Israel does not have a Public Health Minister
	Parliamentary Committees				√		√		√		Proposed legislation will be brought before committees and the subject of obesity has been in committees of child protection and in the health and welfare committee.
	Interdepartmental committees and units	√	√	√	√	√	√	√		√	There is an especially high level of collaboration in the Health Ministry’s Public Health Services, between departments such as Health Promotion, Nutrition and Workplace Health
	Mega-ministries and mergers										Israel has not combined ministries
	Joint budgeting		√	√	√	√	√	√		√	Two main examples: In municipalities, the Ministries of Health and Culture & Sport operate from a shared budget to which both have contributed. The Ministries of Health and Education do so, as well, to promote health-promoting schools. In both of these areas, collaboration is particularly strong on all sides
	Delegated financing		√	√		√	√	√		√	Delegated Funding defines the municipalities program. The National Program’s ministries help create local health promotion infrastructure and require specific process measures, but each municipality designs its own program and allocates funding as it sees fit. Several of the NGO programs involve delegated funding, as well.
	Public engagement	√		√	√	√	√			√	The social marketing program is rooted in public engagement
	Stakeholder engagement			√	√		√				Legislation like calorie-labeling at restaurants and banning advertisements of unhealthy foods during children’s television is conducted in collaboration with stakeholders like the restaurateurs’ union and television networks
	Industry engagement		√	√							The salt program is dependent upon industry engagement, and is built off of a successful program from the UK

From planning through implementation and evaluation, the collaborative nature of the National Program remains one of its defining characteristics. The Health Ministry’s “Healthy Israel 2020” planning process was coordinated by interdepartmental units in the health sector – doctors, nurses, health promoters and academics [29]. These units enabled diverse perspectives with regard to setting goals and targets as well as policy guidelines. The choice to share leadership with the Ministries of Education and Culture and Sport reflect the ministry’s recognition of the fact that successful intersectoriality is, in part, a function of the extent to which non-health sectors own a stake in the process.

The Government’s Social and Economic Affairs Cabinet adopted the National Program, defining active, healthy lifestyle as a societal goal shouldered by the whole of government. This set the legal mandate for joint-planning and budgeting, and laid groundwork for the financial support needed to implement a program of the desired scope. Budget commitments, mostly by the Health Ministry but with sums committed by the Ministries of Education, Culture and Sport, Finance and Agriculture bind these ministries toward working together to achieve this shared goal.

The Ministries of Health, Education and Culture and Sport share the burden of evidence support, setting goals and targets, coordination, policy guidance, implementation and management. Several ministers have provided leadership: The Deputy Minister of Health diverted unprecedented budgets to public health and health promotion and appeared at several launch events for participating municipalities. The Minister of Education pushed the law to ban junk foods from schools through parliament. The Minister of Culture and Sport led legislation to make health clubs more accessible. The Minister of Agriculture provided financial support as well as strong advocacy for the fresh fruit and vegetables scheme at schools.

The inter-ministerial steering committee and working groups are governance structures which formalize health governance, as recommended in HiAP literature. The National Program includes several examples of budget-sharing between ministries, such as the “Municipalities” program and health-promoting schools.

One principle of HiAP is for non-health sectors to adopt health as their own goal. Recognizing the inherent value of healthy students as well as understanding their role in ensuring health-promoting school environments, the Ministry of Education has adopted the slogan “Health is Education.” It has placed promoting students’ health on their permanent list of ministerial objectives. The health sector, in this context, is a steward of health in the service of the Ministry of Education. The Ministry of Education speaks of its school-based health promotion initiatives as its own, a position fully supported by the Ministry of Health.

Legislative progress has been both facilitated and delayed by stakeholder engagement. For example, the requirement for calorie-labeling at restaurants has come up against restaurateurs’ fears of the cost and complexity of calorie-labeling. In addition to working alongside the Ministry of Trade, Labor and Industry, the Director General of the Ministry of Health has worked with the Israeli Union of Restaurateurs to allay fears and facilitate professional guidance on calorie-labeling. An additional example: To support repealing the requirement to obtain a doctor’s permission to join a gym and at the request of the Minister of Culture and Sport, the Health Ministry obtained letters from the national councils of family doctors, pediatricians and sports doctors, declaring the safety of the suggested change.

The Ministry of Culture and Sport recently conducted the largest nationwide survey of physical activity in Israel’s history, in collaboration with the Health Ministry’s Center for Disease Control. In this case, as well, the health sector acted in service of another sector, to the benefit of both and in the name of contributing baseline evidence for evaluation. Other examples include supporting, and at times advancing, other ministries’ projects. The ban on advertising junk food during children’s programming on TV is an example: The Ministry of Communication had previously pursued a voluntary initiative with network television. When efforts stalled, the Health Ministry stepped in. Both ministries are currently working with networks and their public councils to integrate a ban into the networks’ ethical codes.

The social marketing program is fueled by public engagement, via both social media and community-based social marketers. Their role is to coordinate programming with local stakeholders such as parents’ associations, city councils, religious leaders and other local organizations, in order to boost program effectiveness as well as to guide policy, catalyze advocacy, facilitate smoother implementation and management and deepen both monitoring and evaluation.

The evaluation committee is a crucial governance structure, ensuring that evaluation meets current research standards and utilizes Israel’s leading health researchers, their students and the international networks of which they are a part.

The salt and flour enrichment programs rely on industry engagement. To increase the likelihood of success, industry members have been present throughout the planning process, informing goals and targets and means of implementation and management.

Finally, Health in All Policies requires leveraging windows of opportunity and entry points. The National Program has benefited from several, including a priority shift in the Ministry of Health toward public health, the Ministry of Culture and Sport’s desire to expand its scope beyond competitive sports and an Education Ministry with increasing experience in health promotion and a growing willingness to collaborate.

### Remaining steps toward health in all policies

The majority of the National Program’s content is initiated, paid for and implemented by the Ministry of Health. This may be inevitable; it is, after all, a health promotion program. But as discussed above, HiAP entails addressing determinants of health. By leveraging additional ministries’ budgets and policy spheres and intensifying the use of the governance mechanisms described in the WHO framework, the National Program may increase its impact on determinants of obesity and chronic disease outside of its traditional boundaries. While aspects of the National Program resonate with Health in All Policies, the following additional steps could strengthen the health sector’s intersectoral potential and increase the likelihood of fulfilling HiAP’s promise.

A first step: strengthening the intersectoral steering committee. The committee was designed to guide planning and implementation – to lead the National Program. In practice, it became a forum for status updates between members of the Ministry of Health. The mostly low-level representatives from other ministries played a passive role, contributing only when the discussion turned to their specific area of expertise. This must change if the Ministry of Health expects to affect change on a systematic level and catalyze large-scale action on the part of the other ministries involved.

One potential objective is policy coherence, to strive toward a cross-governmental agenda that is conducive, or at the very least, not counterproductive to citizens living an active, healthy lifestyle. The Ministry of Health sits on national and local committees on subjects like food imports, agriculture, meal-services in schools and homes for the aged, urban planning and workplace safety. For example, ministry representatives ensure that hygienic and environmental health standards are considered in urban planning and land development. These memberships can be leveraged in order to coordinate policies which protect and promote healthy lifestyle. More broadly, ministry members can utilize their presence in such forums to defend values which promote population-wide health, like equity, access to services, community, environmental justice, employment and fair housing.

There is, of course, a counterpoint: health professionals already fulfill critical roles in society. Whether out of a sense of pragmatism or professional modesty, it may be best for them to stick to what they know. But more than a decade of research on social determinants of health reveals the extent to which social factors impact health, and gives the health sector a unique perspective on the importance of equitable distribution of resources and protection of rights. This perspective can complement the perspectives of others defending similar values. It may be critical, though, to train members of the health sector to work effectively with professionals from other sectors, in order to provide them with the practical tools to fulfill HiAP.

Knowledge translation is an additional direction. In order to further mobilize other sectors, health expertise can be accessible to and disseminated toward non-health sectors, as well as synthesized and framed according to their language, policymaking contexts and needs [13]. Several countries have implemented Health Impact Assessment (HIA) and Health Equity Impact Assessment (HIEA) toward this end [9]. These tools allow other sectors to understand how their actions affect or will affect health. Israel has not yet adopted assessment tools in policy areas which influence lifestyle. Negev et al [35] present HIA as a platform for facilitating collaboration between the health and environmental sectors in Israel, a vital example worth studying, and perhaps, implementing in other sectors, as well. An additional, related direction could include adding academic representatives from outside of health to the evaluation committee, in order to ensure that data and conclusions are geared toward the sectors upon which the National Program is leaning. Finally, the Health Ministry releases an annual “Minister’s Report” on the state of smoking. It may be beneficial to release a yearly report on the state of nutrition and physical activity, as well.

Ginsberg and Rosenberg’s [26] cost-benefit analysis of several of the National Program’s components is valuable in Israel and abroad. Follow-up study could include breaking down the data according to the sectors whose budgets will be most directly impacted by the National Programs’ components. In addition, policy proposals may be more acceptable at high policymaking levels if issues are expressed as systematic or market failures like externalities, monopolies and information asymmetries, all of which have societal consequences beyond health.

Ollila [13] cites the need to anticipate policy needs and political realities in other sectors, and to be ready when windows of opportunity open. This demands that health professionals step out of the “health box” and become acquainted with other policymaking environments. Doing so would make it more likely to identify additional “win-win” situations between sectors, and strengthen the networks Kickbusch [3] describes as fundamental to HiAP. Ollila [13] articulates the importance of the health sector delving into developing policies in order to identify areas where adverse – or beneficial - health effects are prevalent. Potential overlapping of interests exist, for example, with the Ministry of Agriculture, which represents growers of fruits and vegetables, the Ministry of Environmental Protection, whose broad vision for sustainability shares much in common with public health and the Ministry of Welfare, which is responsible for food security among Israel’s needy.

HiAP literature discusses the need to strengthen collaboration between government and civil society. While the Health Ministry supports several NGO programs, there is not yet a clearly defined and efficiently applied mechanism for galvanizing grassroots health promotion initiatives. In addition, the social protests of summer 2011 revealed that the general public is concerned about social injustice. The depth of research on the social determinants of health, as well as Israel’s health sector’s experience in providing across-the-population access to care, position public health as a potential leader for other sectors amidst public demands for equality and social justice.

While several specific programs feature shared budgets with other sectors, the majority of the National Program’s budget comes from and is managed by the Health Ministry. There is no integrated “National Program Budget,” owned by several sectors. As such, the Health Ministry bears much of the burden of decision-making, management and implementation, which lightens commitments from other sectors. While the National Program is officially a program of three ministries, in practice, it is owned mostly by the Health Ministry. Some in the ministry may see this as an advantage. But if genuine commitment from non-health sectors is a goal, then, over-reliance on the Health Ministry could be a disadvantage.

As mentioned earlier, HiAP sets sights on supra-governmental policy issues like power distribution, poverty and social equity [7]. While the National Program cannot be expected to eliminate poverty, there may be upstream directions to pursue. One example: The value a nation places on health is, in part, a function of the value it places on social welfare. As such, the health sector can play a central role in advancing measurements beyond Global National Product (GNP) to assess national success. Baum and Laris [4] suggest the Happy Planet Index (HPI), which expresses life satisfaction, life expectancy and ecological footprint. The Ministries of Finance and Environmental Protection are currently exploring models like HPI to accompany economic indexes. Promoting such indicators could increase the value the government places on health, and as such, would be worthy pursuits for the National Program.

### A case study in public health intersectoriality

Israel’s national program is an opportunity to study intersectoriality in action. As the program is in its early stages, there is a unique opportunity to design a monitoring effort that can contribute not only to Israel’s program, but, also, to HiAP efforts worldwide. It is an opportunity to learn about points of interaction between health and other sectors. How, why, where and when does each sector respond? What do these “points” look like? Which governance structures in the WHO analytical framework are sustainable and lead to genuine action? How important is informal collaboration, and how are unofficial partnerships affected by being formalized? Are there key elements that mechanisms must include in order for intersectoral action to be successful? What does it even mean for an intersectoral action to be “successful?” Interactions exist throughout the National Program, many of which have the potential to shed light on several of these questions.

Research can be sector-specific (“How can evidence be framed in order to turn the Finance Ministry into an advocate for health promotion?” ”) and general (“To what extent do intersectoral committees facilitate coordination?” “Does joint budgeting lead to more, or less, financial support for health promotion?”). Each sector has its own needs and is entrenched in its own policy narratives. To what extent will the health sector adapt to these needs when doing so may lead to greater health?

An important question is HiAP’s replicability: Can it be implemented anywhere, in any bureaucratic circumstance, and in the long term? There were critical drivers in Israel’s National Program. The Director of the Sports Authority expanded the authority’s focus beyond competitive sports. The Education Ministry’s Health Monitor pushed health promotion to the top echelon of the ministry’s policymakers. And perhaps most importantly, the Health Ministry’s Director General adopted the National Program’s ambitious legislative agenda and continues to protect its budget despite countrywide economic strain. Complementing him is the ministry’s Deputy Director General, who spearheaded “Healthy Israel 2020” and is well-versed in HiAP and participatory approaches to health promotion. How would the program have come together were it not for these individuals? What if there was not such a successful working relationship at the mid-level of the three leading ministries? While there is unmistakable value to leadership, it will be enlightening to understand how dependant HiAP is on the presence of “the right people at the right time.” Israel’s program can be observed over time, to assess if and how partnerships survive after the individuals responsible for launching the program have moved on.

Ollila (2011) recommends identifying policies that represent “win-win” situations for the Health Ministry and others. We can assume that “win-win” policies are most feasible when benefits outweigh barriers for stakeholders and assets to the Health Ministry outweigh risks. But how will policymakers in the Ministry of Health deal with policies whose risks outweigh assets or barriers outweigh benefits? For example, in a collaboration where the Health Ministry can benefit but the stakeholder poses risks – for example, working with the food industry – will the ministry take steps to avert the risks? Regarding a policy where a stakeholder faces a major barrier – for example, the education system losing resources by halting the sale of junk food – will the Health Ministry alter the offered policy package to enhance benefits to the stakeholder or compensate for the barrier? It will also be possible to assess which assets and risks are valuable to the Health Ministry, and which barriers and benefits impact the various stakeholders.

It is important to study the inter-ministerial steering committee. Will it continue to be a venue for the Health Ministry to update the others, or will it catalyze genuine leadership? To what extent will the committee’s recommendations be implemented? The same questions may be asked with regard to other working groups.

Studying the evolution of the evaluation committee can be valuable, as well. Israel’s public health academic community and members of the Ministry of Health have a longstanding history of collaboration. How will the panel address the tension between academic integrity and the immediacy of the task at hand?

Initiatives like the salt and flour fortification programs, where progress is dependent upon industry, have taken a long time to materialize. At times, they have stalled entirely. This is due, of course, to the independence and often competing interests of industry representatives. These and other points of coordination between the Health Ministry and private stakeholders are opportunities for process evaluation, to assess at what rate and under what conditions progress is made.

Finally, Health Ministry/health sector workers involved in intersectoral aspects of the National Program will no doubt be impacted. How much time will they spend engaged in work outside of their traditionally-defined roles? Will intersectoral work distract from competing responsibilities? How will they respond to policy needs of other sectors? How will they evaluate the intersectoral experience? Will the Health Ministry equip its workers with the skills and knowledge to engage beyond health? Addressing these issues can offer insight into the feasibility of Health in All Policies.

## Conclusion

Israel’s National Program to Promote Active, Healthy Lifestyle is an intersectoral, inter-ministerial approach to address Israel’s obesity epidemic and its resultant burden on chronic disease. This paper has explored Israel’s National Program according to the Health in All Policies (HiAP) framework for horizontal and vertical governance in health, aimed at addressing the social determinants of health. The paper’s objective was twofold: One, to identify where Israel’s National Program both reflects and falls short of HiAP, and, two, to assess ways in which the National Program could be utilized as a case-study in HiAP and public health intersectoriality.

As mentioned in the article, elements of the National Program, such as joint planning, integration in the policy agendas and settings of other ministries and budget-sharing, to a more limited extent, adhere to the principles of HiAP. Israel would increase its HiAP potential by strengthening these and other directions, including utilizing the inter-ministerial steering committee to lead the National Program, leveraging the Health Ministry’s widespread presence in and out of government, and focusing on knowledge translation and dissemination according to the policy needs and knowledge bases of other sectors.

HiAP, as mentioned above, may exact a price. Expecting health professionals to leave traditional tasks with which they are already overburdened for unchartered policy terrain, will surely affect their personal and professional effectiveness and wellbeing. Clearly, there is a need to strike a balance. While this article does not discuss what this balance might be, by analyzing an HiAP-like program in action and outlining potential directions for further study, it is our hope that HiAP’s utility, on the one hand, and its limits, on the other, will become more clear.

## Appendix 1

### Implementation of health in All policies

Finland pioneered intersectoriality in the 1970s and formally introduced HiAP in 2006 [4]. The country continues to be a global leader in intersectoral health governance; 75-120 structures between the years 2008 and 2011 included representatives from the Ministry of Social Affairs and Health and other ministries [36]. The WHO recently published examples of countries, states and cities around the world that are attempting aspects of Health in All Policies. The following section draws on these examples as well as the 2012 edition of *Eurohealth: Quarterly of the European Observatory on Health Systems and Policies*[37].

Eastern European governments have crossed sectors in order to control consumption of tobacco products. In Ukraine, the Finance Ministry has spearheaded a tax increase on tobacco products. Tax revenue has increased and smoking rates have decreased by 13% [38]. Moldova, as well, has increased taxes on tobacco products [39]. In Serbia, the Tobacco Control Strategy and the Action Plan for Tobacco Control include contributions from Trade and Services, Environment, Mining and Spatial Planning, Agriculture, Forestry and Water Management, Justice, Labor and Social Affairs, Culture, Internal Affairs, Education, Finance and Youth and Sport [40].

Several countries have utilized intersectoriality to address nutrition. In Albania, the Ministries of Agriculture, Food and Consumer Protection, Finance, Education and Science, Labour and Social Assistance and Equal Opportunities committed in 2010 to create a joint decision-making structure and adopt a national intersectoral Food and Nutrition Action Plan [41]. In Cyprus, The Ministries of Health, Agriculture, Natural Resources and Environment, Commerce, Industry and Tourism and Education and Culture are addressing food safety and nutrition [42].

Governments are utilizing existing intersectoral cabinets and committees: Northern Ireland’s Executive Committee of the Assembly emphasizes cross-community support and cross-departmental cooperation, allowing for health considerations to inform decision-making [43]. In South Australia, Excomm, the Executive Committee of Cabinet, mandates the health sector to contribute in areas such as migration, digital technology, water security, children’s literacy and drivers licensing [44].

Other countries address health needs via other ministries. In Cyprus, the Ministry of Interior authorizes health services for immigrants and asylum seekers, while the Ministry of Education and Culture facilitate screening, immunization and health education in schools [42]. “Mega-ministries” attempt to coordinate cross-sectoral action, as well. In 2010, Hungary formed the Ministry of National Resources, including health, education, welfare, culture and sports. Health officials are utilizing the opportunity to catalyze inter-departmental actions for health [45].

Newly formed governance structures facilitate intersectoral action for health, too. In 2004, France introduced the Public Health National Committee, including representatives from health, social affairs, education, universities, security, defense, justice, economics, agriculture and environment and the national health insurance service. In Slovakia, a committee with representation from the Ministry of Transport, Post and Telecommunications as well as Ministries of Internal Affairs, Finance, Defense, Justice, Education and Science, Environment, Health and Construction and Regional Development, led a successful reduction in road traffic accidents and related fatalities in 2008-9 [46]. In 2010, California created the Health in All Policies Task Force, becoming the first state in the US to adopt HiAP. The task force promotes healthy lifestyle via active transportation, housing, parks and green spaces and improving access to healthy, affordable foods. Recommendations include removing barriers to institutional acquisition of locally grown produce, adding health to planning and transportation criteria and adopting assessment tools to project long-term costs and benefits of proposed legislation [47].

Additional countries are making strides toward HiAP. In addition to the examples listed above, Shankardass et al [7] list Brazil, Cuba, England, Iran, Malaysia, New Zealand, Norway, Quebec, Scotland, Sri Lanka, Sweden, Thailand and Wales.

## Competing interests

The authors have no competing interest to declare.

## Authors’ contributions

YK conducted the literature review and drafted the manuscript. IG, RW, ND, DM and YF guided and provided significant insight throughout all analytical and editorial stages. All authors read and approved the final manuscript.

## Authors’ information

Yannai Kranzler, MA, is a National Health Promoter for the Public Health Services in the Israel Ministry of Health, and a Doctoral Student in the Faculty of Health Sciences at Ben Gurion University of the Negev.

Nadav Davidovich, MD, PhD, is an Associate Professor in the Faculty of Health Sciences at Ben-Gurion University of the Negev.

Yonina Fleischman is a National Health Promoter for the Public Health Services in the Israel Ministry of Health, who will be receiving her MPH from the Hebrew University in June 2013.

Itamar Grotto, MD, PhD, is the Director of the Public Health Services in the Israel Ministry of Health. He is also an Associate Professor at Ben-Gurion University of the Negev.

Daniel S. Moran, PhD, is the Manager of the National Program to Promote Active, Healthy Lifestyle. He is also a Professor in the Faculty of Health Sciences at the Ariel University Center of Samaria.

Ruth Weinstein, MPH, is the Head of the Department of Education and Health Promotion in the Public Health Services of the Israel Ministry of Health.
